# Notes and News

**Published:** 1893-02-25

**Authors:** 


### Notes and News.

COLLECTED AND COMMUNICATED.

The Royal Commission on Pensions for the Aged Poor are carrying forward their work with great energy. The subject is one of great importance and general interest. The Prince of Wales was present at one of the most recent meetings of the Committee, and himself took part in the proceedings.

Mr. Conrad W. Thies will read a paper on "The London Poor and their Medical Needs" before The Hospitals Association on Wednesday, March 22nd, at the Royal Free Hospital, Gray's Inn Road, when Mr John Hutton, Chairman of the London County Council, will take the chair at eight p.m.

The humble individual bids fair to be lost in the vast arrangements at Chicago. Naturally the existing accommodation would be quite inadequate to receive the many visitors who will flock hither. A mammoth hotel is being erected by the World's Fair Co-operative Bureau. The hotel is constructed in blocks, somewhat after the plan of St. Thomas's Hospital, and will contain over 6,000 rooms. It is stated that these rooms will be let at a uniform rate of a dollar a day to those who pay a nominal registration fee beforehand, and thus secure the right of occupancy at any time during the exposition. At the City Press Agency, 51, Coleman Street, E.C., all information respecting this City within walls can be obtained.

A reduction in the number of beds at the Charing Cross Hospital is again threatened. In so populous a locality as that in which the hospital is situated this necessity would, indeed, be a calamity, and yet unless very substantial aid is soon forthcoming this will perforce take place. There is a limit to the extent to which a deficit is permissible, and the £13,000, which it appears will have to be placed on the wrong side of the accounts for the ensuing year, is too serious a sum to be viewed with aught but apprehension. We regret to learn that Mr. Passmore Edwards' most generous and useful gift for a convalescent home has not been yet applied, owing to difficulties attending the proposed site.

A costly and beautiful gift has been received by the Pope from Prince Luitpold, the Regent of Bavaria. This present, which is in commemoration of the Jubilee of his Holiness, consists of a model of the column of the Virgin, the original of which is in the Marien Platz at Munich. It is five feet high, of massive gold, costly diamonds being set in the Virgin's crown; whilst sapphires, rubies, and emeralds ornament the pedestal.

The Lambeth Workhouse, Renfrew Road, had a very distinguished visitor last week, when the Prince of Wales paid the establishment a visit, inspected all the wards and arrangements for the sick and the poor inmates. H.R.H. is not only a member, in name, of the Aged Poor Commission, but is a sympathetic and an active member besides. After the Royal visitor had seen the serving of the dinners in the hall and in the wards, and complimented the managers on the excellence of all the arrangements, he made the following entry in the visitors' book: "Visited this house to-day, and found everything most clean and in excellent order.—Albert Edward P."

The annual meeting of the Manchester and Salford Life-Boat Saturday Council and Committee took place last week. The Mayor presided, and Mr. Alexander Muir, the Hon. Treasurer, read the statement of accounts, showing that £4,680 had been raised by the Life-Boat Saturday collections. Mrs. Macara, Hon. Secretary of the Ladies' Council, read the statement of accounts of the Ladies' Fund, which amounts to £902. These sums do not include the amount from the Manchester Branch Committee, the two together being nearly £5,000. The people of Manchester and Salford have reason to be proud that they have a second time contributed a sum towards the support of the Royal National Life-Boat Institution, which, although not equal to that of the year before, is, nevertheless, a noble example to the whole country. Bolton last year raised the handsome sum of over £1,300. Stockport has had a second Life-Boat Saturday, and has raised a sum very similar to the previous year. Preston, Bury, Warrington, and Runcorn had their first Life-Boat Saturdays last year, having all raised very creditable amounts. Various other places followed in the movement, which has spread to Scotland. The first Life-Boat Saturday there was organised by Mr. C. W. Miller, Secretary of the Sailors and Firemen's Union at Dundee, and the handsome sum of £900 was raised. Public meetings are being held for the furtherance of Life-Boat Saturday in a number of places. An immense demonstration is also being organised for the East and West Ridings of Yorkshire, to take place on July 6th, and to be an annual fixture. Mr. Macara, the originator of Life-Boat Saturday, referred during the meeting to the part the ladies had taken in the movement during the past year. He said that there is no doubt that what had been achieved by them was a wonderful success, and that they, by their great perseverance and systematic working, had shown the way by which a national institution can be supported, even during a time of commercial depression, and without injuring any other charity. Over 250 ladies have taken part in this work, and they have succeeded in getting contributions from 10,000 people at least. Already the example of the ladies of Manchester and Salford has been followed in other towns. In every respect the Life-Boat Saturday is worked with energy, and we wish Mr. Macara and the movement every success.

